# PNPLA3 I148M mediates the regulatory effect of NF‐kB on inflammation in PA‐treated HepG2 cells

**DOI:** 10.1111/jcmm.14839

**Published:** 2019-12-03

**Authors:** Shuhua Yuan, Hongxia Liu, Ding Yuan, Jing Xu, Yunzhi Chen, Xiao Xu, Fen Xu, Hua Liang

**Affiliations:** ^1^ Department of Endocrinology and Metabolism Guangdong Provincial Key Laboratory of Diabetology The Third Affiliated Hospital of Sun Yat‐Sen University Guangzhou China; ^2^ Department of Endocrinology Guangzhou Panyu Central Hospital Guangzhou China; ^3^ Department of Endocrinology The First Affiliated Hospital of Zhengzhou University Zhengzhou China; ^4^ Department of Endocrinology and Metabolism The Affiliated Hospital of Guizhou Medical University Guiyang China

**Keywords:** ER stress, inflammation, NAFLD, NF‐kB, PNPLA3

## Abstract

Both PNPLA3 I148M and hepatic inflammation are associated with nonalcoholic fatty liver disease (NAFLD) progression. This study aimed to elucidate whether PNPLA3 I148M is involved in NF‐kB‐related inflammation regulation in NAFLD. HepG2 cells homozygous for the PNPLA3 I148M mutation were used. The human PNPLA3 promoter sequence was screened for NF‐kB binding sites using the MATCH and PATCH tools. NF‐kB‐mediated transcriptional regulation of the PNPLA3 gene was assessed by luciferase reporter assay, EMSA and ChIP‐qPCR. Wild‐type (I148I) and mutant (M148M) PNPLA3 were overexpressed using stable lentivirus‐mediated transfection. The pCMV vector and siRNA were transiently transfected into cells to direct NF‐kB overexpression and PNPLA3 silencing, respectively. A putative NF‐kB binding site in the human PNPLA3 promoter was shown to be necessary for basal and NF‐kB‐driven transcriptional activation of PNPLA3 and protein/DNA complex formation. Supershift analysis demonstrated a protein/DNA complex specifically containing the NF‐kB p65 and p50 subunits. ChIP‐qPCR confirmed the endogenous binding of NF‐kB to the human PNPLA3 promoter in response to NF‐kB overexpression and palmitic acid (PA) challenge. The silencing of PNPLA3 blocked the overexpression of NF‐kB or PA‐induced TNF‐α up‐regulation. Moreover, mutant PNPLA3 overexpression prevented NF‐kB inhibitor–induced down‐regulation of TNF‐α expression in PA‐treated HepG2 cells. Finally, the overexpression of mutant but not wild‐type PNPLA3 increased TNF‐α expression and activated the ER stress–mediated and NF‐kB‐independent inflammatory IRE‐1α/JNK/c‐Jun pathway. Human PNPLA3 was shown to be a target of NF‐kB, and PNPLA3 I148M mediated the regulatory effect of NF‐kB on inflammation in PA‐treated HepG2 cells, most likely via the IRE‐1α/JNK/c‐Jun ER stress pathway.

## 1 INTRODUCTION

Nonalcoholic fatty liver disease (NAFLD) is a clinicopathological syndrome characterized by diffuse ballooning fatty degeneration of hepatocytes and fat storage and excludes effects caused by alcohol and other known harmful factors to the liver.^1^ The histological spectrum of NAFLD ranges from simple steatosis to steatohepatitis, hepatic fibrosis, cirrhosis and even hepatocellular carcinoma.^1^, ^2^ The histologic progression of NAFLD involves two steps of liver injury (the two‐hit theory): The first hit is mainly intrahepatic fat accumulation caused by insulin resistance, and the second hit is mainly liver injury and steatohepatitis caused by inflammation.^3^

Genome‐wide association studies (GWAS) have shown that the PNPLA3 gene, which is mainly expressed in the liver and fat, is susceptible to NAFLD.^4^ The function of the PNPLA3 gene remains unclear, although in vitro studies have shown that the PNPLA3 protein has triacylglycerol (TG) hydrolase,^5^ lysophosphatidyl acyltransferase (LPAAT)^6^ and calcium‐independent phospholipase A2 (iPLA2) activities.^7^ The PNPLA3 I148M (rs738409) polymorphism is associated with not only liver fat content but also hepatocyte steatohepatitis, fibrosis and cirrhosis,^8^ suggesting that PNPLA3 I148M plays a key role in the progression of NAFLD. As the two‐hit theory of NAFLD claims that inflammation contributes to the progression of NAFLD and more inflammatory infiltration and liver damage were found in NAFLD patients carrying PNPLA3 I148M than those carrying wild‐type genotype,^9^ we speculated that PNPLA3 I148M is closely related to liver inflammation. Furthermore, NF‐kB is the most important transcription factor regulating inflammation and involved in the pathogenesis of NAFLD progression.^10^ Therefore, we are very interested in the involvement of the PNPLA3 gene in NF‐kB signalling, which links inflammatory responses in NAFLD. In this study, we show that PNPLA3 is a target gene regulated by NF‐kB and that the PNPLA3 M148M protein participates in regulating the palmitic acid (PA)–induced inflammatory response in HepG2 cells carrying a homozygous PNPLA3 148M genotype. These findings may provide a new means to elucidate the role of PNPLA3 I148M in NAFLD.

## 2 METHODS

### 2.1 Cell culture and treatment

Human hepatocellular carcinoma (HepG2) cells obtained from cell bank of CAS were cultured in MEM/EBSS medium (SH30024.01B, Hyclone) supplemented with 10% foetal bovine serum (10099‐141, Gibco). HepG2 cells at 60%‐70% confluence were starved in serum‐free medium overnight before treatment. To induce FFA overloading, the cells were treated with 500 mmol/L palmitic acid (PA, P5585, Sigma‐Aldrich) for certain times. Control cells were incubated with the same medium containing the same amount of solvent (BSA) used to dissolve the PA. For treatment experiments, the cells were pretreated with 10 μmol/L PDCT (P8765, Sigma‐Aldrich) for 6 hours before transfection or 10 μmol/L BAY11‐7082 (B5556, Sigma‐Aldrich) for 1 hour before PA treatment.

### 2.2 Construction of plasmid vectors

To construct pGL3‐WT luciferase reporter vector, the 542 nt fragment (−506 to +36) of human PNPLA3 gene containing putative binding sites for NF‐kB was amplified by PCR using the following KpnI/MluI site‐linked primers: sense 5′‐ATGGTACCATCAAGTGGGCACCAACTG‐3′ and antisense 5′‐ AT ACGCGT GAAGGACAAGCTCCAGCC‐3′, and was cloned in the pGL3‐Basic firefly luciferase reporter vector (Promega). Site‐directed mutagenesis of the putative NF‐kB target‐site (GCGCGGGGAGCTCCCA → GCGTGGGCCTCCCGCC) with the pGL3‐WT vector as the template was performed using the MutanBEST kit (D401, Takara) according to the manufacturer's instructions and named PGL3‐Mutant. The expression vector NF‐kB p65 (pCMV‐P65) was kindly provided by Professor Jianping Ye from Department of Pennington Biomedical Research Center, Louisiana State University. The pCMV‐IκBαM vector was commercially purchased (631923, Takara), which contains a mutated form of IκBα with a serine‐to‐alanine mutation at residues 32 and 36 resulting in failure of signal‐induced phosphorylation. All plasmid DNAs were extracted and purified with EZNA Endo‐free Plasmid Midi Kit (D6915‐03, Omega Bio‐Tek) and sequenced (Invitrogen).

### 2.3 Transient transfection and Dual‐luciferase reporter assay

The pGL3‐WT and PGL3‐Mutant luciferase reporter vectors were transfected alone or each cotransfected with pCMV‐p65 plasmid into HepG2 cells using X‐tremeGENE HP DNA Transfection Reagent (06366236001, Roche). Cells transfected with the pGL3‐Basic vector or empty pCMV (pCMV‐Mock) vector were used as control. Cells were lysed 24 hours post‐transfection in passive lysis buffer (Promega). Firefly and Renilla luciferase signals were measured by the Dual‐Luciferase ® Reporter Assay System (Promega) on an Infinite F500 microplate reader (Tecan). Three biological replicates were done for each sample in luciferase assays. Normalized reporter activity was expressed as the firefly luciferase value divided by the Renilla luciferase value (RLA), which was then normalized to the control vector activity.

### 2.4 SiRNA (small interfering RNA) transfection

Human PNPLA3 was knocked down using siRNA oligoribonucleotides targeting PNPLA3 (SiRNA‐PNPLA3) with the following target sequences: GTGACAACGTACCCTTCAT. A non‐targeting siRNA (SiRNA‐Mock) (5′‐ UUCUCCGAACGUGUCACGU TT 3′) was used as a control. HepG2 cells were treated with 2 μg siRNA using X‐tremeGENE siRNA Transfection Reagent (04476493001, Roche) in a serum‐free medium for 6 hours, and then, the medium was supplemented with serum and maintained in culture for 48 hours. Cells were then either lysed to use for Western blotting or were evaluated for mRNA expression.

### 2.5 Electrophoretic Mobility Shift Assay (EMSA)

Nuclear extracts were prepared from HepG2 cells using Nuclear Extraction Kit (Panomics, 13938). Double‐stranded oligonucleotides used as probes comprising the putative NF‐kB binding site of human PNPLA3 (sense: 5′‐AGTCGCTGCGGGGAGCTCCCAGGCT GGACCC‐3′) were end‐labelled with IRD 700. The Odyssey Infrared EMSA Kit (829‐07910, LI‐COR) was used to examine protein‐DNA‐binding activity in extracts and labelled probes. For the competition experiments, unlabelled competitors containing wild‐type (WT cold) or mutant (Mut. cold: 5′‐AGTCGCTGCGAAACTTCAACAGGCTGGACCC‐3′) putative NF‐kB binding site were added at 200‐fold excess. For supershift analysis, anti‐p50 and anti‐p65 antibodies (sc‐53744 and sc‐71675, Santa Cruz Biotechnology) were incubated with the nuclear extracts prior to the addition of labelled probes. An anti‐SREBP‐1c antibody was used as negative control for supershift analysis.

### 2.6 Chromatin immunoprecipitation (ChIP)‐qPCR assay

Chromatin was prepared from HepG2 cells treated with pCMV‐Mock, or pCMV‐p65, or pCMV‐Mock+PA. Chromatin was enzymatically sheared and immunoprecipitated with anti‐NF‐kB p65 antibody (sc‐71675, Santa Cruz Biotechnology) using Pierce™ Agarose ChIP Kit (26156, Thermo Fisher Scientific) in accordance with the kit instructions. Rabbit IgG was used as a mock antibody for negative control. Immunoprecipitated chromatin was subjected to real‐time qPCR with the SYBR Premix DimerEraser (Perfect Real Time) (RR091A, Takara). Two ChIP‐qPCR primer pairs that overlap the NF‐kB binding site of the human PNPLA3 promoter region are designed as follows: sense1 5′‐TGGGCACCAACTGCGACTCC‐3′, sense2 5′‐ GCAGTGTCTGCTGGAGTT TTGG‐3′; Shared antisense 5′‐CGATGGCGGAGGA CCTGTG‐3′. Samples were run in triplicate, and data from NF‐kB IP and control IP were presented as enrichment relative to input DNA. ChIP‐qPCR was repeated twice to confirm the reproducibility of results. The quality of chromatin enzymatically sheared was assessed using agarose gel electrophoresis (Figure S1A). Dissolution curve of the primers is presented in Figure S1B.

### 2.7 Isolation of total RNA extraction and analysis by qPCR

Total RNA was extracted from HepG2 cells using TRIzol reagent (Invitrogen) according to the manufacturer's instructions. The mRNA expression was assessed by real‐time‐PCR analyses using the SYBR Premix DimerEraser (Perfect Real Time) (RR091A, Takara) on a LightCycler® 480 System (Roche Applied Science). The relative expression values were calculated relative to β‐actin by using the 2−ΔCT method. The data shown in the study represent the average of three independent experiments. The oligonucleotide primers used in this study are shown in Table S1.

### 2.8 Western blotting and ELISA

Total cell lysates were extracted from HepG2 cells using cell lysis buffer (PH 6.8, 50 mmol/L Tris, 1% SDS, protease inhibitor). Nuclear protein was extracted using NucBuster™ Protein Extraction Kit (71183‐3, Novagen). Protein concentration was detected by BCA method. Boiled cell lysates were subjected to SDS‐PAGE separation on 8%‐12% gels and transferred to polyvinylidene fluoride (PVDF) membranes (Millipore). Primary antibodies against PNPLA3 (ab81874, Abcam), NF‐kB p65 (ab4764, Abcam), SREBP‐1 (ab3259, Abcam), SP3 (sc‐644, Santa Cruz), TNF‐α (3707, Cell Signaling Technology), Bip (3183, Cell Signaling Technology), c‐Jun (9165, Cell Signaling Technology), IRE‐1α (3294, Cell Signaling Technology), JNK2 (9258, Cell Signaling Technology), pJNK (Cell Signaling Technology), or β‐actin (4970, Cell Signaling Technology) were purchased commercially. Antimouse or anti‐rabbit fluorescent secondary antibodies were used, and the bands were visualized using the Odyssey infrared fluorescence imaging system (LI‐COR). The arachidonic acid (AA) levels in cell culture supernatants were measured using an ELISA kit (OKEH02583, Aviva Systems Biology) according to the manufacturer's instructions.

### 2.9 Construction of lentiviral‐based stable HepG2 cell lines overexpressing PNPLA3

Human PNPLA3 M148M was initially PCR amplified from HepG2 using the primer sequences: F 5′‐TGCTCTAGAGCCACCATGTACGACGCAGAGCGCGGC TGG‐3′; R 5′‐GAATGCGGCCGCTCACAGACTCTTCTCTAGTGAAAAAC‐3′ (restriction sites underlined). The amplicon was then digested with Xbal and Notl and cloned by ligation into PCDH‐CMV‐EF1a‐GFP‐puro lentivector. Ligation reaction was then added to thawed competent DH5a *E coli* cells and incubated reaction on ice, and then heat shock the competent cell mixture; the competent cells were added with LB and shaken. After centrifugation, supernatant was removed and precipitate was suspended in the remaining solution for spreading onto LB plates with ampicillin. White clones were selected and inoculated into LB liquid medium and shaken. The positive clones were selected for plasmid extraction, PCR and electrophoresis, and further identified by sequencing. For the construction of lentivector PNPLA3 I148I, lentivector PNPLA3 M148M was used as the template and two synthetic oligonucleotide primers containing the desired mutation with each complementary to the opposite strands of the vector are extended during temperature cycling by Pfu DNA Polymerase. After PCR, the lentivector M148M was removed by digestion with Dpn I, and the lentivector I148I was remained in the reaction. The mutagenic primers are as follows: F 5′‐CCTGCTTCATCCCCTTCTACAGTGG CCTTATCCCTC‐3′; R 5′‐GTAGAAGGGCATGAAGCAGGAACATACCAAGG‐3′ (mutation sites underlined). PCDH‐PNPLA3‐I148I and PCDH‐PNPLA3‐M148M lentivector were verified by sequencing (Figure S2). Finally, HepG2 cells were transferred with lentivector‐empty (LV‐Mock), lentivector‐PNPLA3 M148M (LV‐148M) and lentivector‐PNPLA3 I148I (LV‐148I) and the PNPLA3 expression was detected by real‐time PCR. The oligonucleotide primers for real‐time PCR are shown in Table S2.

### 2.10 Statistical analysis

Data represent the mean ± standard deviation (SD) values of three independent duplicate experiments. Statistical analysis was performed using one‐way ANOVA analysis of variance followed by Student's *t* test. A value of *P* < .05 was considered to be statistically significant.

## 3 RESULTS

### 3.1 NF‐kB is involved in regulating human PNPLA3 expression

**Figure 1:**
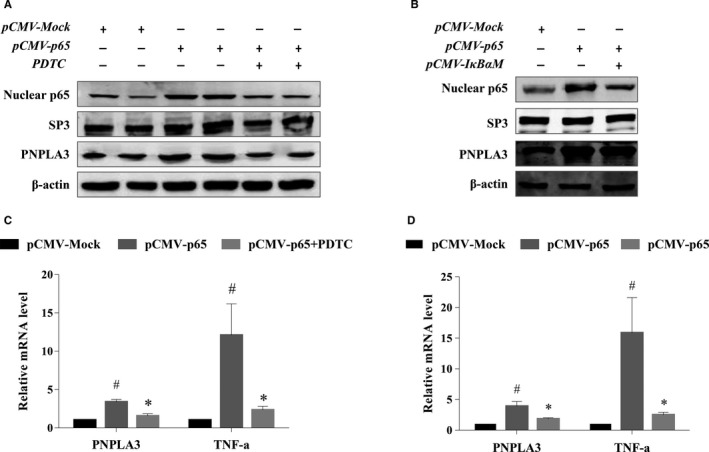
PNPLA3 expression was regulated by NF‐kB in HepG2 cells. HepG2 cells were transfected with blank pCMV (pCMV‐Mock) or pCMV‐p65 with or without pretreatment of NF‐kB inhibitor PDTC for 6 h, and then, the protein expression of PNPLA3 and nuclear NF‐kB (A), and mRNA expression of PNPLA3 and TNF‐α (C) were detected using Western blotting and real‐time PCR, respectively. HepG2 cells were transfected with pCMV‐Mock or pCMV‐p65 with or without pre‐transfection of pCMV‐IkBαM, and then, the protein expression of PNPLA3 and nuclear NF‐kB (B), and mRNA expression of PNPLA3 and TNF‐α (D) were detected using Western blotting and real‐time PCR, respectively. The results of real‐time PCR are presented as relative mRNA levels from three independent experiments normalized to the mock transfected control. #*P* < .05 compared with pCMV‐Mock, *P < .05 compared with pCMV‐p65

To clarify the relationship between NF‐kB and PNPLA3 expression, we detected the expression of PNPLA3 protein and mRNA in HepG2 cells transiently overexpressing NF‐kB p65. As shown in Figure 1, the protein (Figure 1A,B) and mRNA (Figure 1C,D) levels of PNPLA3 were significantly higher in NF‐kB p65‐overexpressing HepG2 cells than in the mock controls. However, this increase in PNPLA3 expression induced by NF‐kB overexpression was blocked when cells were pretreated with pyrrolidine dithiocarbamate (PDTC) (Figure 1A,C) or transfected with pCMV‐IκBαM (Figure 1B,D). The change in the mRNA expression of PNPLA3 was consistent with that of TNF‐α, a well‐known target gene of NF‐kB (Figure 1C,D).

### 3.2 Identification of a NF‐kB binding site −357/−366 bp upstream of the translation start site of the human PNPLA3 gene

**Figure 2:**
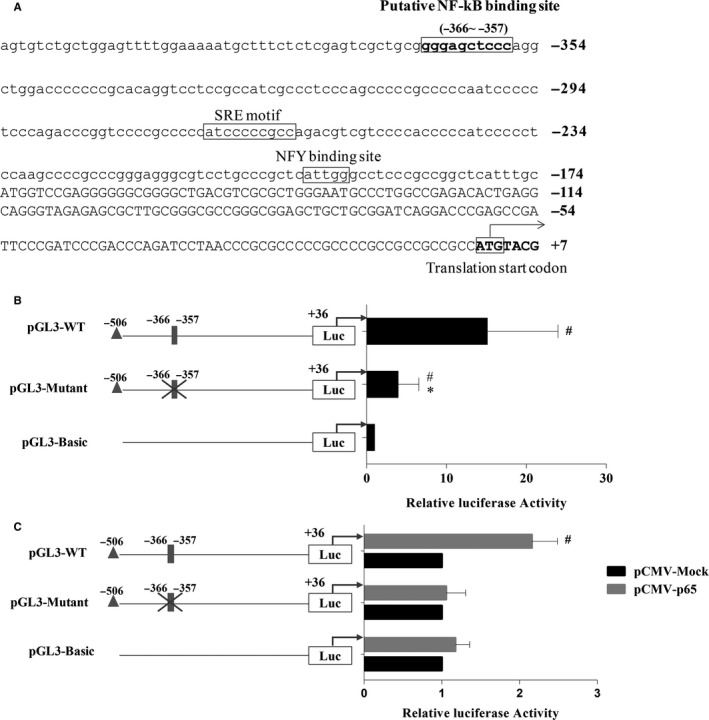
NF‐kB transactivated human PNPLA3 promoter through a putative NF‐kB binding site. A, Human PNPLA3 promoter upstream of the 5′UTR. The putative NF‐kB binding sites are highlighted with boxes; SREBP‐1c and NFY binding sites are underlined with a thick line. The ATG translation start codon where the A is numbered with 1 is indicated in bold and boxed. B, Relative luciferase activity of different PNPLA3 promoter‐reporter constructs. HepG2 cells were transfected with pGL3‐WT, PGL3‐Mutant and pGL3‐Basic reporter constructs for 24 h to measure the relative luciferase activities of PNPLA3 promoter by dual‐luciferase assays. Relative luciferase activity was corrected for Renilla luciferase activity and normalized to the pGL3‐Basic activity from three independent experiments. #*P* < .05 compared with pGL3‐Basic, *P < .05 compared with pGL3‐WT. C, NF‐kB‐driven relative luciferase activities from different PNPLA3 promoter‐reporter constructs in HepG2 cells. Each PNPLA3 promoter‐reporter construct was transiently cotransfected with pCMV‐Mock or pCMV‐p65 into HepG2 cells. Cell lysates were collected 24 h post‐cotransfection, and dual‐luciferase assays were performed. Relative luciferase activity was corrected for Renilla luciferase activity and normalized to the pCMV‐Mock activity from three independent experiments. # *P* < .05 compared with pCMV‐Mock. A grey‐filled rectangle represents the putative NF‐kB binding site, and a cross represents the mutation of the binding element

The Matrix Search for Transcription Factor Binding Sites (MATCH)^11^ and Pattern Search for Transcription Factor Binding Sites (PATCH) programs were used to search for NF‐kB binding sites up to 5.0 kilobases upstream of the human PNPLA3 promoter. The TRANSFAC 9.4 database^12^ was used to construct specific binding site weight matrices for prediction. We found a putative NF‐kB binding site located −357 bp to −366 bp upstream of the translation start site of human PNPLA3 (Figure 2A) that conforms well to the NF‐Kb binding consensus sequence (GGG RNN YYC C, where R is A or G, and Y is C), although DNA sequence alignment analysis showed this sequence was not conserved with mouse, rat and chicken (Figure S3).

The pGL3‐WT, PGL3‐Mutant and pGL3‐Basic reporter constructs were transiently transfected into HepG2 cells, and basal transcriptional activity was measured 24 hours after cotransfection by dual‐luciferase reporter assay. As shown in Figure 2B, the relative luciferase activity in pGL3‐WT‐transfected cells was 15‐fold that of pGL3‐Basic‐transfected cells (*P* < .05), while the relative luciferase activity in PGL3‐Mutant‐transfected cells was reduced by 70% compared with that in pGL3‐WT‐transfected cells (*P* < .05), suggesting the critical role of the putative NF‐kB binding site in maintaining basal transcriptional activity of the PNPLA3 promoter.

To determine whether the putative NF‐kB binding site affects NF‐kB‐driven transcriptional activation of PNPLA3, the pGL3‐WT and PGL3‐Mutant reporter vectors were cotransfected with the pCMV‐Mock or pCMV‐p65 vectors into HepG2 cells. NF‐kB p65‐induced transcriptional activity was detected by dual‐luciferase reporter assay. As shown in Figure 2C, luciferase activity in cells transfected with pCMV‐p65 was 2.4‐fold higher than that in cells transfected with pGL3‐WT (*P* < .05) but pCMV‐p65 transfection had no effect on the activity of cells transfected with PGL3‐Mutant. These results suggested that the putative NF‐kB binding site is essential for NF‐kB‐driven transcriptional activation of PNPLA3.

**Figure 3:**
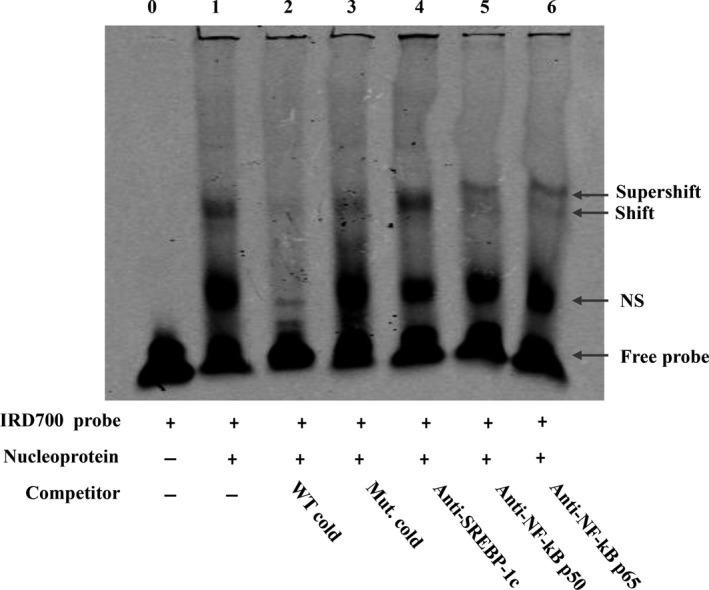
NF‐kB bound to the human PNPLA3 promoter. EMSA was performed using an IRD‐700‐labelled double‐stranded probe containing the putative NF‐kB binding sequence of human PNPLA3 gene promoter. The incubation of nuclear protein extracted from HepG2 cells and labelled probe was performed without or with the indicated unlabelled oligonucleotides (WT cold probe or Mut. cold probe) in the competition assays. And a supershift assay was performed to specifically assert the DNA‐protein interactions by using NF‐kB p65 and p50 antibody. SREBP‐1c antibody was used as control for supershift assay. Lane 1: no competitor; Lane 2: WT cold competitor (200×); Lane 3: mutant cold competitor (200×); Lane 4: SREBP‐1c antibody; Lane 5: NF‐kB p50 antibody; Lane 6: NF‐kB p56 antibody. The result is representative of two independent experiments with similar results

Electromobility shift assay (EMSA) was further conducted to clarify whether NF‐kB interacts with the human PNPLA3 promoter through the putative NF‐kB binding site. Nucleoprotein extracted from HepG2 cells was found to bind to a probe containing the wild‐type putative NF‐kB binding site of the human PNPLA3 promoter (Figure 3, lane 1). Protein/probe binding could be blocked by a high concentration of unlabelled wild‐type probe (Figure 3, lane 2) but not a high concentration of unlabelled mutant probe (Figure 3, lane 3). The addition of both anti‐NF‐kB p50 and anti‐NF‐kB p65 antibodies to the EMSA binding reaction induced a supershift band (Figure 3, lane 6 and lane 7), but no supershift band or reduction in band intensity was observed in the presence of anti‐SREBP‐1c antibody (Figure 3, lane 5).

### 3.3 PA‐induced PNPLA3 expression was mediated by NF‐kB

#### 3.3.1 NF‐kB, rather than SREBP‐1c, regulated PNPLA3 expression in long‐term PA‐treated HepG2 cells

**Figure 4:**
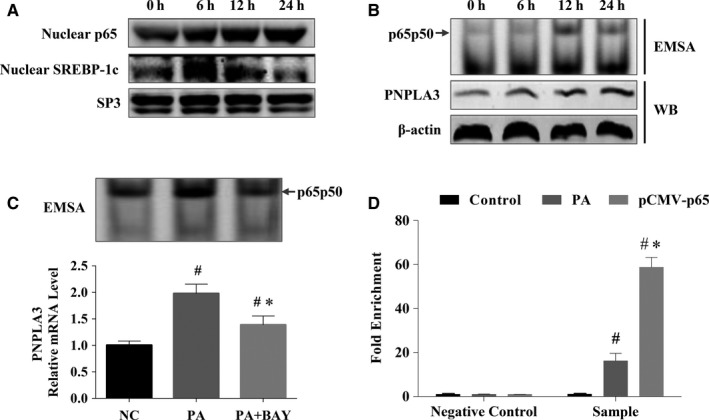
Binding of NF‐kB to human PNPLA3 promoter mediated expression of PNPLA3 during long‐term PA treatment. A, Nucleoprotein expression trait of NF‐kB and SREBP‐1c with time during PA treatment. Nucleoprotein levels of NF‐kB and SREBP‐1c were measured at 0 h and 6 h, 12 h, and 24 h post‐treatment of PA by Western blotting and normalized to SP3; B, PA increased nucleoproteins binding to PNPLA3 promoter containing the putative NF‐kB binding site. Nucleoproteins and cytoplasmic proteins were extracted from HepG2 cells at 0 h and 12 h and 24 h post‐treatment of PA. Nucleoproteins incubated with the IRD‐700‐labelled double‐stranded DNA (dsDNA) probe of human PNPLA3 promoter containing the putative NF‐kB binding site. The binding of protein and probe was detected by EMSA. The cytoplasmic PNPLA3 protein expression was detected by Western blotting; C, BAY‐117082 attenuated the binding of nucleoprotein extracted from HepG2 cells treated with PA for 24 h and probe of human PNPLA3 promoter containing the putative NF‐kB binding site. Nucleoproteins and mRNA were extracted from HepG2 cells 24 h post‐treatment of PA with or without pretreatment of BAY‐117082. The binding of nucleoproteins and the dsDNA probe of human PNPLA3 promoter containing the putative NF‐kB binding site was detected by EMSA. PNPLA3 mRNA level was detected by real‐time PCR. Results of real‐time PCR are presented as relative mRNA levels from three independent experiments normalized to the control. #*P* < .05 compared with NC, *P < .05 compared with PA D, PA increased the endogenous binding of NF‐kB and PNPLA3 promoter in vivo. The endogenous binding of NF‐kB and PNPLA3 promoter was detected by CHIP‐qPCR, which was performed using an anti‐NF‐KB P65 antibody and chromatin prepared from HepG2 treated with PA for 24 h or pCMV p65 for 24 h. Fold enrichment as percentage of input DNA was calculated. Rabbit IgG was used as a mock antibody for negative control in ChIP. #*P* < .05 compared with control; *P < .05 compared with PA

Given that SREBP‐1c is a known PNPLA3 target gene that can also be activated by PA, it is first necessary to clarify changes in SREBP‐1c and NF‐kB expression over time during PA intervention to avoid confounding effects of SREBP‐1c. Expression of nucleoprotein NF‐kB and SREBP‐1c was detected before (0 hours) and after 6, 12 and 24 hours of PA treatment. As shown in Figure 4A, the nucleoprotein expression of NF‐kB gradually increased between 6 hours and 24 hours of PA treatment, while the nucleoprotein expression of SREBP‐1c peaked after 6 hours of PA treatment and then decreased gradually over time until it returned to the level observed before intervention after 24 hours. This finding suggests that NF‐kB rather than SREBP‐1c plays a transcriptional regulatory role during long‐term (24 hours) PA treatment.

The interaction of nucleoprotein NF‐kB p65 and the PNPLA3 promoter, and simultaneous PNPLA3 protein expression were detected before and after 6, 12 and 24 hours of PA treatment. Nucleoproteins extracted from HepG2 cells treated with PA were hybridized with fluorescently labelled PNPLA3 promoter probes containing the putative NF‐kB binding site and used to conduct EMSA experiments. As shown in Figure 4B, the binding of NF‐kB and the PNPLA3 promoter probe began at 12 hours of PA treatment, and this presence of binding was still detectable at 24 hours of PA treatment. PNPLA3 protein expression was increased beginning after 6 hours of PA treatment and further increased at 12 hours and 24 hours. These results suggested that PNPLA3 expression after 6 hours of PA treatment can be attributed to the transcriptional activation of SREBP‐1c, while PNPLA3 expression after 24 hours of PA treatment should be attributed to the transcriptional activation of NF‐kB.

**Figure 5:**
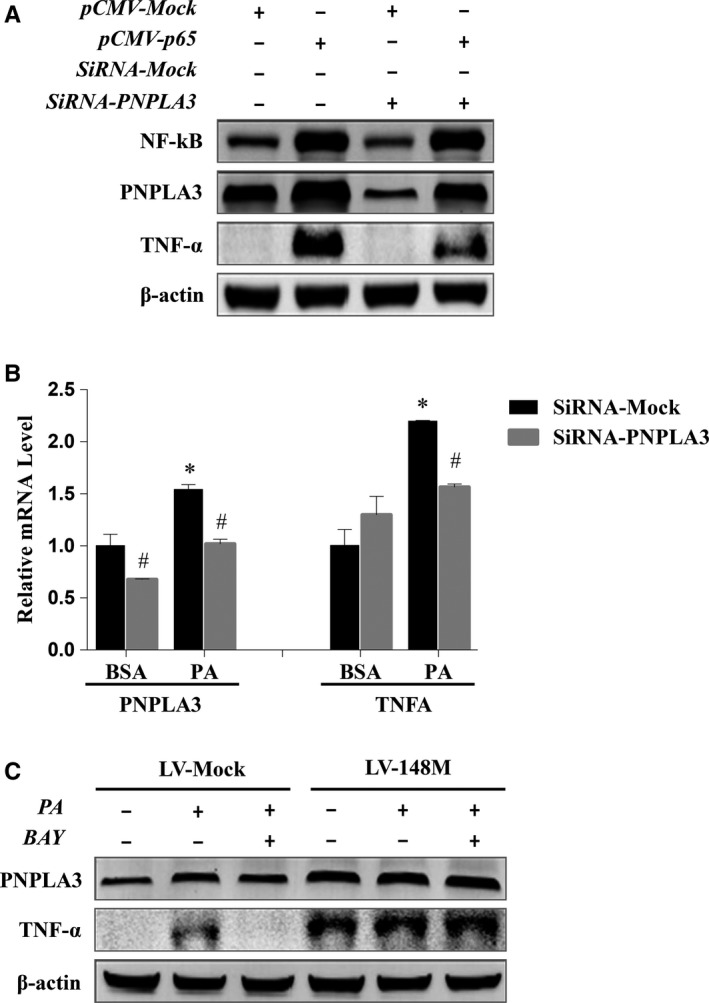
PNPLA3 mediated the NF‐kB regulation of inflammation in PA‐treated HepG2 cells. A, PNPLA3 mediated NF‐kB regulation of TNF‐α expression in HepG2 cells. HepG2 cells were transfected with pCMV‐p65, or cotransfected with pCMV‐p65 and siRNA‐PNPLA3. Cells transfected with the pCMV‐Mock or/and siRNA‐Mock were used as negative control. Cell lysates were collected 24 h post‐transfection. The protein expressions of NF‐kB, PNPLA3 and TNF‐α were detected by Western blotting. B, Inhibition of PNPLA3 alleviated PA‐induced hepatocyte inflammation. HepG2 cells were treated with PA for 24 h with pre‐transfection of siRNA‐Mock or siRNA‐PNPLA3. The mRNA levels of PNPLA3 and TNF‐α were detected by real‐time PCR. The results are presented as the mean ± SD from three independent experiments. #*P* < .05 compared with siRNA‐Mock‐transfected cells, *P < .05 compared with siRNA‐Mock‐transfected cells treated with BSA; C, Overexpression of PNPLA3 M148M prevented NF‐kB inhibitor‐induced reduction of PA‐related inflammation. HepG2 cells that stably transfected with LV‐148M or LV‐Mock were treated with PA for 24 h with or without pretreatment of BAY‐117082. Cell lysates were harvested to measure the protein expressions of PNPLA3 and TNF‐α by Western blotting

To further verify that NF‐kB mediated the effect of long‐term PA treatment on PNPLA3 expression, we pretreated HepG2 cells with the NF‐kB specific inhibitor BAY11‐7082 (BAY) and then detected the effect of 24 hours of PA treatment on the interaction between NF‐kB and the PNPLA3 promoter as well as simultaneous PNPLA3 expression. As shown in Figure 5C, PA markedly enhanced the binding of NF‐kB to the PNPLA3 promoter and significantly increased the PNPLA3 mRNA level by 2‐fold (*P* < .001). In addition, BAY almost completely inhibited PA‐induced binding of NF‐kB and PNPLA3 and significantly decreased PA‐induced PNPLA3 expression by approximately 1.4‐fold (*P* = .003) (Figure 4C). This result suggested that long‐term PA treatment increases the expression of PNPLA3 by NF‐kB.

#### 3.3.2 PA increases the endogenous binding of NF‐kB to the human PNPLA3 promoter

The endogenous interaction between NF‐kB and the PNPLA3 promoter was detected by ChIP‐qPCR in HepG2 cells treated with PA for 24 hours. Cells overexpressing NF‐kB due to pCMV NF‐kB p65 plasmid transfection were used as a positive control. As shown in Figure 4D, the putative NF‐kB target region was enriched by almost 16‐fold (*P* < .05) over the negative control following PA treatment and almost 60‐fold when NF‐kB was overexpressed (*P* < .05). These findings suggested that PA increases the endogenous binding of NF‐kB and the PNPLA3 promoter and that PNPLA3 is regulated directly by NF‐kB in vivo.

### 3.4 PNPLA3 mediated the inflammatory regulation by NF‐kB in PA‐treated HepG2 cells

To determine the role of PNPLA3 in NF‐kB‐mediated inflammatory regulation, HepG2 cells were transfected with pCMV‐Mock or pCMV‐p65 vector with control or target siRNA (si‐Mock or si‐PNPLA3, respectively). Transfection with pCMV‐p65 induced the marked overexpression of NF‐kB and up‐regulation of PNPLA3 and TNF‐α; however, the up‐regulation of TNF‐α induced by pCMV‐p65 transfection was alleviated after the partial silencing of PNPLA3 with siRNA (Figure 5A), suggesting that PNPLA3 mediates the regulatory effect of NF‐kB signalling on expression of the inflammatory factor TNF‐α in HepG2 cells.

To determine whether PNPLA3‐mediated regulation of inflammation by NF‐kB is involved in the pathogenesis of NAFLD, we first evaluated the effect of PNPLA3 inhibition on TNF‐α mRNA expression in an NAFLD cell model induced by PA overloading. As shown in Figure 5B, PNPLA3 and TNF‐α mRNA levels were increased by 1.5‐ and 2‐fold, respectively, in response to PA treatment. SiRNA‐PNPLA3 significantly inhibited PNPLA3 gene expression (*P* < .05), which significantly alleviated PA‐induced TNF‐α production (*P* < .05), although knockdown of PNPLA3 did not reduce TNF‐α expression to baseline levels observed in HepG2 cells without PA treatment. This finding suggests that PNPLA3 is involved in inflammatory regulation in PA‐treated HepG2 cells.

We next examined the effects of PNPLA3 overexpression on TNF‐α expression in PA‐treated HepG2 cells treated with an NF‐kB inhibitor to elucidate whether PNPLA3 regulates TNF‐α expression mediated by NF‐kB in NAFLD. Given that the HepG2 cell line carries PNPLA3 M148M, we used lentivirus to construct a HepG2 cell line stably overexpressing PNPLA3 M148M for experiments. Consistent with the qPCR results shown in Figure 5B, PA treatment increased PNPLA3 protein expression, and the NF‐kB inhibitor BAY reduced PA‐induced PNPLA3 expression in control cells (LV‐Mock) (Figure 5C). Similarly, the increase and decrease in TNF‐α expression induced by PA and an NF‐kB inhibitor, respectively, were observed in LV‐Mock‐transfected cells. In cells overexpressing PNPLA3 M148M (LV‐148M), TNF‐α expression under basal and PA treatment conditions was increased compared with that in control cells; however, BAY did not inhibit the increase in TNF‐α expression induced by PA. These results suggested that PNPLA3 M148M mediates the regulatory effect of NF‐kB on inflammation in PA‐treated HepG2 cells.

### 3.5 **PNPLA3 M148M, but not wild‐type PNPLA3, regulated TNF‐**α **expression and activated the ER stress–associated IRE‐1α/JNK/c‐Jun pathway**

**Figure 6:**
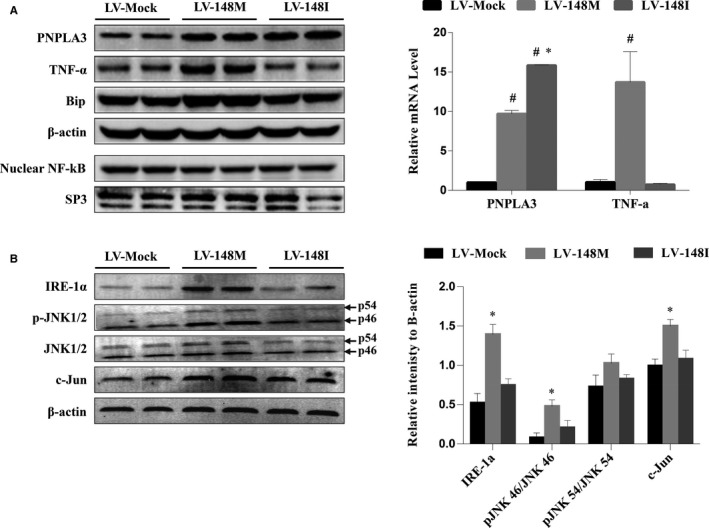
Overexpression of PNPLA3 M148M activated ER stress signal IRE‐1α‐JNK‐c‐Jun inflammatory pathway. A, PNPLA3 M148M overexpression increased TNF‐α expression in a NF‐kB independent way. HepG2 cells were stably infected with lentiviral PNPLA3 M148M (LV‐148M), lentiviral PNPLA3 I148I (LV‐148I) and mock lentivirus (LV‐Mock), respectively. Nucleoprotein and cytoplasmic protein were extracted after infection to measure protein expressions of nuclear NF‐kB, PNPLA3 and TNF‐α by Western blotting (left panel). RNA was extracted to measure PNPLA3 and TNF‐α mRNA levels by real‐time PCR (right panel). The real‐time PCR results are presented as the mean ± SD from three independent experiments. #*P* < .05 compared with LV‐Mock; *P < .05 compared with LV‐148M. B, PNPLA3 M148M but not I148I overexpression activated IRE‐1α‐JNK‐c‐Jun pathway. HepG2 cells were stably transfected with LV‐148M, LV‐148I and LV‐Mock, respectively. Cytoplasmic protein was extracted to measure protein expressions of IRE‐1α, total and phosphorylation JNK1/2, and c‐Jun by Western blotting (left panel). The relative band intensities (right panel) in Western blots (n = 2) were determined using ImageJ and normalized to β‐actin. Statistical significance was performed by one‐way ANOVA. Data are presented as the mean ± SD. *P < .05 compared with LV‐Mock

As PNPLA3 I148M is a well‐known risk genotype for NAFLD, while those with PNPLA3 I148I are protected from NAFLD, we sought to determine whether these two genotypes mediate different inflammatory responses. TNF‐α expression levels in lentiviral‐based HepG2 cell lines stably overexpressing PNPLA3 M148M (LV‐148M) and I148I (LV‐148I) were compared. The results (Figure 6A) showed an increase in both TNF‐α mRNA and protein expression in LV‐148M but not LV‐148I cells. This observation was not attributed to a difference in iPLA2‐related inflammation between LV‐148M and LV‐148I cells as they expressed similar levels of arachidonic acid (AA) (4.72 ± 2.18 and 3.87 ± 1.7 ng/mL, respectively), an inflammation‐related product of iPLA2 catalysis. Interestingly, nuclear NF‐kB expression did not change in either LV‐148M or LV‐148I cells, suggesting an NF‐kB‐independent mechanism of inflammation regulation in PNPLA3 M148M cells. In addition, the endoplasmic reticulum (ER) stress marker Bip was increased in only LV‐148M cells, which drove us to further examine the IRE‐1α/JNK/c‐Jun pathway, a known NF‐kB‐independent inflammatory pathway induced by ER stress. As shown in Figure 6B, we found the significant up‐regulation of IRE‐1α, phosphorylated‐p46/p46 JNK isoform, and c‐Jun expression, but no significant effect on the level of phosphorylated‐p54/p54 JNK isoform, in LV‐148M cells. No significant change in levels of expression of these factors was found in LV‐148I cells.

## 4 DISCUSSION

The PNPLA3 I148M is susceptible to NAFLD^4^ and NAFLD progression,^8^ and this susceptibility has been unequivocally shown to different ethnic group. However, regulation of the PNPLA3 gene and the function of the PNPLA3 protein as well as its role in NAFLD remain largely unknown. Inflammation plays an important role in NAFLD progression; however, there has been no report on whether mutant PNPLA3 protein is involved in inflammatory regulation in NAFLD. In this study, we investigated NF‐kB‐mediated transcriptional regulation of the PNPLA3 gene in PA‐treated HepG2 cells carrying homozygous PNPLA3 M148M genotype and the effect of PNPLA3 M148M protein on inflammation in vitro. The proinflammatory transcription factor NF‐kB transactivated the PNPLA3 gene via targeting a binding site located −357 bp to −366 bp upstream of the translation start site of human PNPLA3. In HepG2 cells treated with PA for 24 hours, PNPLA3 was transcriptionally regulated by NF‐kB rather than SREBP‐1c, and mutant PNPLA3 M148M, but not wild‐type PNPLA3 I148I, was involved in PA‐induced regulation of the inflammatory factor TNF‐α. This study is the first to report the mechanism by which NF‐kB regulates PNPLA3 and the role of PNPLA3 I148M in regulating inflammation in NAFLD.

Although PNPLA3 is the most studied gene susceptible to NAFLD, a functional study using knockout mice did not find any abnormal metabolic phenotype.^13^, ^14^ Moreover, liver‐specific PNPLA3 I148M overexpression^15^ and I148M point mutation knock‐in mice^16^ exhibited significant susceptibility to liver fat deposition induced by a high‐sucrose diet but did not show evidence of the promotion of NAFLD progression, such as nonalcoholic steatohepatitis (NASH) and hepatic fibrosis. Phenotypic differences between murine models and human patients suggested the limitations of murine models in studying PNPLA3. The different PNPLA3 protein sequence lengths and gene regulation between human and mouse have been shown; the human PNPLA3 protein is 97 amino acids longer than the murine PNPLA3 protein at the C‐terminal end, which contains two‐vesicle targeting motifs,^17^ suggesting human‐specific functions of PNPLA3. In addition, ChREBP, a lipogenic glucose‐sensing transcription factor, was found to transcriptionally regulate PNPLA3 gene expression in mice and not humans.^18^ In previously identified mouse and human sterol regulatory elements (SREs),^19^, ^20^ in addition to the conserved SRE, human and mouse PNPLA3 gene had their novel non‐conserved SRE, respectively (Figure S3), suggesting different intrinsic mechanisms of SREBP‐1c‐mediated regulation of PNPLA3 gene between them. The observation that human NF‐kB binding site was not conserved with mouse in the current study indicates the uniqueness of NF‐kB in regulation of human PNPLA3, which might account for the lack of inflammation in the livers of mouse models. Thus, it is difficult for murine models to mimic all the biological functions of human PNPLA3. The HepG2 cell line, which is widely used to study liver diseases, was found to carry homozygous PNPLA3 M148M genotype.^17^ This characteristic makes HepG2 cells a natural mutation model in which to study the function of human PNPLA3 I148M and its role in NAFLD. Some researchers even consider HepG2 cells to be an ideal in vitro model for studying PNPLA3.^17^

SREBP‐1c is a transcription factor that regulates lipid synthesis and can also be activated by PA in vitro. Our previous study showed the direct transcriptional regulation of human PNPLA3 by SREBP‐1c.^19^ Therefore, in addition to the effect of NF‐kB on PNPLA3 expression, the effect of SREBP‐1c on PNPLA3 expression after PA treatment should be considered. In this study, the nuclear expression of SREBP‐1c and NF‐kB varied with PA treatment time. The pattern of changes in SREBP‐1c and NF‐kB expression was consistent with a report from Nagaya et al^21^ showing that inflammation persisted, and hepatic lipid deposition and SREBP‐1c expression decreased as NASH progressed. Based on our results, 24 hours was chosen as the PA treatment time because the interaction between NF‐kB and PNPLA3 was significant at this time, and nuclear SREBP‐1c expression was close to its baseline level.

GWAS found that people carrying PNPLA3 I148M are susceptible to the presence and progression of NAFLD,^8^ but the mechanism of this susceptibility is still unknown. Whether increased susceptibility is attribute to a functional gain due to increased LAPPT activity^6^ or a functional deficiency due to inhibited TG lipase activity^5^, ^22^ remains controversial. Additionally, the mechanism by which PNPLA3 I148M promotes NAFLD progression could not be explained by its LAPPT or TG lipase activities. This study in a PA‐induced NAFLD HepG2 cell model is the first to report that PNPLA3 I148M is regulated by NF‐kB and involved in the regulation of TNF‐α expression. TNF‐α is a proinflammatory cytokine that contributes to the second hit in NASH pathogenesis.^23^ Thus, our results provide new insight to elucidate the function of PNPLA3 I148M mutant protein and its role in NAFLD and explain why NAFLD patients carrying the PNPLA3 148M are more susceptible to progressive NAFLD.

The mechanism by which PNPLA3 regulates the inflammatory response is worth discussing. PNPLA3 has mild iPLA2 activity in vitro.^7^ AA and lysophosphatidylcholine (LPC) produced from membrane phospholipids by the enzymatic action of iPLA2 are involved in modulating inflammation. Theoretically, PNPLA3 I148M therefore may regulate TNF‐α expression due to an increase in PNPLA3‐mediated AA production. However, overexpression of wild‐type and mutant PNPLA3 in HepG2 cells in our study or in transgenic mice did not alter the levels of AA, or hepatic AA‐derived prostaglandins or LPC.^13^ Thus, increased iPLA2 activity could not explain the increased proinflammatory effects of PNPLA3 I148M compared to those of wild‐type PNPLA3. ER stress plays a crucial role in NASH progression.^24^ Lipid droplets (LDs) originate from the ER and regulate ER stress.^25^ In both HuH7 cells^26^ and I148M mutation knock‐in mice,^16^ PNPLA3 I148M mutant protein, but not wild‐type PNPLA3, was found to localize mainly in LDs. Therefore, the different distributions of LDs with the expression of wild‐type and mutant PNPLA3 protein might explain why mutant proteins activated ER stress but wild‐type proteins did not. As the overexpression of wild‐type PNPLA3 had no effect on ER stress, the question as to what the role of NF‐kB in regulating wild‐type PNPLA3 in NAFLD is then arises. NF‐kB is involved in regulating the anti‐apoptotic pathway. Recently, Ochi et al^27^ reported that ER stress–related apoptosis induced by TM was increased in mice deficient in PNPLA3, suggesting the anti‐apoptotic effect of wild‐type PNPLA3. Determining whether wild‐type PNPLA3 mediates the anti‐apoptotic effect of NF‐kB requires further study. Finally, our study demonstrated the close relationship between PNPLA3 I148M and ER stress–related inflammation. IRE‐1α/JNK/c‐Jun is a key ER stress–related inflammatory signalling pathway independent of NF‐kB that participates in TNF‐α‐induced hepatocyte injury and apoptosis.^28^ Activation of the JNK/c‐Jun pathway and increased proinflammatory cytokines in human primary I148M hepatic stellate cells (HSCs) have been reported,^29^ which is consistent with our results. The central role of JNK in the development of steatohepatitis was recently revealed, and JNK is a potential therapeutic target for steatohepatitis. Our findings might provide new hope for the accurate treatment of NAFLD in patients carrying PNPLA3 I148M.

**Figure 7:**
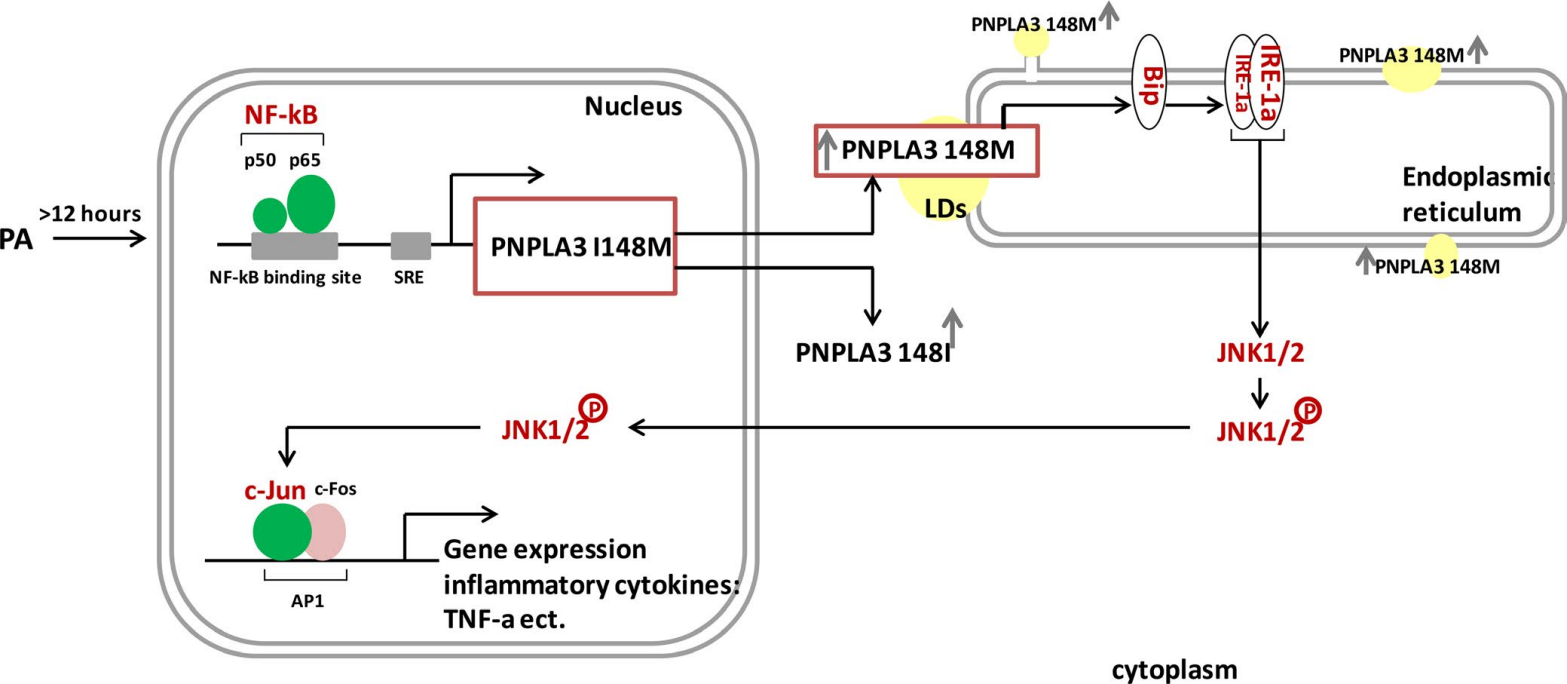
Scheme of PNPLA3 I148M‐related inflammatory signalling in HepG2 treated with PA. PNPLA3 gene is transcriptionally up‐regulated by NF‐kB during long‐term PA treatment. The distribution of wild‐type and mutant PNPLA3 proteins is different: I148M mutant proteins are distributed in lipid droplets, while wild‐type proteins are distributed in the cytoplasm. PNPLA3 I148M protein then activates the IRE1a signalling of ER stress, followed by phosphorylating JNK1/2 and up‐regulating c‐Jun expression, which finally up‐regulates c‐Jun‐dependent expression of inflammatory cytokines, such as TNF‐α

In summary, as shown in Figure 7, we found that the human PNPLA3 gene is a target of NF‐kB and contains an NF‐kB‐binding site −357 bp to −366 bp upstream the PNPLA3 translation start site. In addition, PNPLA3 I148M was shown to be transcriptionally activated by NF‐kB to increase TNF‐α expression through the ER stress IRE‐1a/JNK/c‐Jun pathway in HepG2 cells treated long‐term with PA.

## CONFLICT OF INTEREST

The authors declare that they have no conflict of interest.

## AUTHOR'S CONTRIBUTIONS

The authors' responsibilities were as follows: L.H conceived and designed the experiments and drafted the manuscript. Y.‐SH and L.‐H.X performed the experiments. YD, C.‐YZ and XX analysed the data. XF interpreted the data. All the authors gave their final approval to the submitted and published versions.

## Data Availability

The data that support the findings of this study are available from the corresponding author upon reasonable request.
