# Supplementary material for: PNPLA3 I148M mediates the regulatory effect of NF‐kB on inflammation in PA‐treated HepG2 cells
Source: J Cell Mol Med. 2019 Dec 3;24(2):1541–52. doi: 10.1111/jcmm.14839 (PMC6991629; doi:10.1111/jcmm.14839)
Supplement: Supplementary file 1 [file JCMM-24-1541-s001.docx]

**Supplementary Data**

**Table S1. Primers for qPCR target genes**

|  | | Sequences |
| --- | --- | --- |
| β-actin | Sense | 5'-CTCCATCCTGGCCTCGCTGT-3' |
|  | Anti-sense | 5'-GCTGTCACCTTCACCGTTCC-3' |
| PNPLA3 | Sense | 5'-AATGTCCACCAGCTCATCTC-3' |
|  | Anti-sense | 5'-TCACTCACTCCTCCATCCAC-3' |
| TNF-α | Sense | 5'-GTGAGGAGGACGAACATC-3' |
|  | Anti-sense | 5'-GAGCCAGAAGAGGTTGAG-3' |

**Table S2. Primers for validation of PNPLA3 overexpression in HepG2 cell lines transfected stably with LV- 148M and LV-148I**

|  | | Sequences | |
| --- | --- | --- | --- |
| β-actin | Sense | | 5'-ACAGAGCCTCGCCTTTGCCGAT-3' |
|  | Anti-sense | | 5'- CTTGCACATGCCGGAGCCGTT-3' |
| PNPLA3 I148I | Sense | | 5'- CCTTGGTATGTTCCTGCTTCA-3' |
|  | Anti-sense | | 5'- ACACGGTGATGGTTGTTTTGG -3' |
| PNPLA3 M148M | Sense | | 5'- CCTTGGTATGTTCCTGCTTGA -3' |
|  | Anti-sense | | 5'- ACACGGTGATGGTTGTTTTGG -3' |

**Legends**

**FIGURE S1 A, Efficient shearing of chromatin.** Chromatin samples are enzymatically sheared, de-crosslinked and analyzed on a 2% agarose gel. M: DNA makers, from top to bottom were 2000bp, 1000bp, 750bp, 500bp, 250bp, 100bp, repectively; 1: Control; 2: PA; 3: pCMV-p65; **B, Dissolution curve of the primers.** Left panel: PNPLA3 amplification curve, Right panel: PNPLA3 melting curve.

**FIGURE S2 Sequencings of PCDH-PNPLA3-I148I and PCDH-PNPLA3-M148M.** Red line: amino acid coden 148 of PNPLA3; Arrow: variant nucleotide of PNPLA3 I148M.

**FIGURE S3 Multiple alignment of human, rat, mouse and chicken PNPLA3 promoters.** Sources of DNA sequence are from Ensembl database：ENST00000216180.7 for human, ENSMUST00000045289.5 for mouse, ENSRNOT00000015767.7 for rat, and ENSGALT00000073442.2 for chicken. Sequences were aligned using the online Clustal Omega program (<https://www.ebi.ac.uk/Tools/msa/clustalo/>). The putative NF-kB binding site is boxed in black. The previously identified mouse and human transcription factor binding sites are double underlined. Shading regions indicate consensus between human, mouse and rat; and * indicates that the nucleotide in that position is conserved in all four species. The numbers refer to the translation start codon (underlined with wavy line) where the A is numbered with 1. SRE: sterol regulatory element; ChoRE: carbohydrate response element.

**Fig.S1**

**Fig. S2**

**Fig. S3**
